# Public Transport Accessibility for People With Disabilities: Protocol for a Scoping Review

**DOI:** 10.2196/43188

**Published:** 2023-03-28

**Authors:** Claudel R Mwaka, Krista L Best, Stéphanie Gamache, Martine Gagnon, François Routhier

**Affiliations:** 1 Center for Interdisciplinary Research in Rehabilitation and Social Integration Québec, QC Canada; 2 Rehabilitation Department Université Laval Québec, QC Canada; 3 Library Université Laval Québec, QC Canada

**Keywords:** accessibility, public transport, disability, experience, self-efficacy, satisfaction, scoping review

## Abstract

**Background:**

Transportation is essential for people of all ages and backgrounds to live a fulfilling and satisfying life. Public transport (PT) can facilitate access to the community and improve social participation. However, people with disabilities may encounter barriers or facilitators in the whole travel chain that can lead to negative or positive perceptions in terms of self-efficacy or satisfaction. These barriers may be perceived depending on the nature of the disability. Few studies have identified PT barriers and facilitators experienced by people with disabilities. However, findings were focused mainly on specific disabilities. Access requires broader considerations of barriers and facilitators for various types of disabilities.

**Objective:**

This scoping review aims to describe the barriers and facilitators to the use of PT experienced by people with various disabilities in the whole travel chain and to explore perceived experiences, self-efficacy, and satisfaction when using PT.

**Methods:**

A scoping review will be conducted using Arksey and O’Malley’s framework and the PRISMA-ScR (Preferred Reporting Items for Systematic Reviews and Meta-Analyses Extension for Scoping Reviews) checklist. The literature search will be conducted using the electronic databases MEDLINE, Transport Database, and PsycINFO via Ovid platform, Embase, and Web of Science from 1995 to 2022. Two reviewers will independently identify studies based on inclusion (published in English or French, outcomes on PT accessibility for people with disabilities, peer-reviewed or guideline reports or editorials) and exclusion (no full text, focused on a technology system, outcome validation study, study on no-fixed route PT accessibility, etc) criteria and extract the data. When a study has addressed the accessibility of multiple modes of PT, including fixed-route PT, it will be retained. However, only data on fixed-route PT will be extracted. Any related systematic reviews identified through the search will be retained, and the reference lists will be hand-searched and screened for inclusion criteria.

**Results:**

The search we performed on July 21, 2022, in the databases mentioned above allowed us to retrieve 6399 citations. Of these citations, 31 articles were identified, and data extraction was performed. As of March 11, 2023, we have started data analysis. The findings will be synthesized narratively to summarize the barriers and facilitators to PT, perceived experiences with PT, self-efficacy for using PT, and satisfaction with PT according to the Human Development Model-Disability Creation Process conceptual framework.

**Conclusions:**

The results of this scoping review could lead to a better understanding of the potential barriers and facilitators to the use of PT by people with various types of disabilities and how negative or positive experiences throughout the travel may influence their self-efficacy and satisfaction. The results may be used to provide recommendations to PT providers and policy makers to work together to make PT accessible, usable, and inclusive for all people with disabilities.

**Trial Registration:**

Open Science Framework OSF.IO/2JDQS; https://osf.io/2jdqs

**International Registered Report Identifier (IRRID):**

DERR1-10.2196/43188

## Introduction

On the basis of the 2010 global population estimates, the World Health Organization (WHO) and World Bank [1] report that more than 1 billion people live with some form of disability, including motor, sensory, and cognitive; intellectual; or mental disabilities. This represents approximately 15% of the world’s population, which is 5% more than a previous WHO estimate from the 1970s. More than 46% of people 60 years and older have disabilities and more than 250 million older adults experience moderate to severe disabilities [2].

Given their disability or aging-related health conditions, many people may experience restrictions in accessing transportation. In transportation modes, public transport (PT) is essential for people of all ages and backgrounds to live a fulfilling and satisfying life. For example, PT plays a vital role in many aspects of daily life including access to employment, education, health care, shopping, social occasions, and multiple recreational activities [3]. Thus, it may facilitate participation in community activities and employment if it is accessible to people with disabilities. PT is a system of vehicles such as buses and trains that operate at regular times on fixed routes and are used by the public [4]. Accessible PT enables people with disabilities to live independent lives and means having access to transport services that are available where and when one wants to travel, being informed about the services, knowing how to use the services, being able to use the services, and having the means to pay for them [5]. This suggests that the accessibility of PT involves more than simply boarding and disembarking. To ensure access, an understanding of all the elements that make up the travel chain is required [6], such as leaving the home to wait for the transport at the stop or station, availability of timetable information, boarding, moving within the transport, disembarking, the use of sidewalks, and the attitudes of drivers and other passengers toward people with disabilities [7]. Thus, people with disabilities may encounter barriers or facilitators related to any element of the travel chain that can lead to negative or positive perceptions in terms of self-efficacy or satisfaction, and these may, in turn, affect their willingness to use PT.

Defined as the belief in one’s ability to perform a specific task [8], self-efficacy has been identified as the most important predictor of travel behavior change [9]. Self-efficacy is determined by factors such as past experiences or accomplishments and emotional reactions [8,10,11]. Past accomplishments that are interpreted as the result of a skill developed in the past [12] have been found to be the most important in influencing self-efficacy [10]. Emotional reactions can improve or reduce self-efficacy. Thus, positive experiences with PT use may generate positive emotions that can enhance feelings of personal efficacy toward PT use. Conversely, negative experiences can induce negative emotional reactions such as anxiety and doubt, which in turn may impact self-efficacy for using PT. Therefore, low self-efficacy may be a barrier to PT use [9].

Satisfaction is described as an intrinsic positive consequence emerging from a behavior that fulfills the expectations of an individual [13]. Satisfying experiences have been shown to increase intrinsic motivation, which increases the likelihood to continue the behavior. Consequently, experiencing satisfaction during interventions is assumed to increase the likelihood that the individual will sustain the behavior change [9]. The most relevant features of the transportation system, such as trip duration, accessibility, fare, network connectivity, information, comfort, safety, and employees’ kindness, may influence user satisfaction. Satisfaction with travel can have a significantly positive effect on the frequency of PT use [14]. Indeed, the more satisfied PT users are with their travel experience, the more they tend to use PT for their work commute [14].

We are interested in all PT modes including buses, trains, tramways, subways, and ferries because people with disabilities travel less frequently and rely on PT more than the general population [15]. Long before the adoption of the United Nations Convention on the Rights of Persons with Disabilities (UN-CRPD) in 2006 [16], many countries including the United States, Canada, and Australia developed regulations, codes, and standards to regulate the practices related to the accessibility of PT [17]. Indeed, in 1990, the Americans with Disabilities Act (ADA) qualified accessible and usable transportation as a qualified civil right [5]. In Australia, the Disability Standards for Accessible PT 2002 (as amended in 2010) were enforced to provide transport operators and providers with information on their obligations under the Disability Discrimination Act (DDA). Under the Canada Transportation Act (1996), the Canadian Transportation Agency was made responsible for removing obstacles to mobility in all federally regulated (air, rail, marine, and interprovincial bus) transportation services and facilities. Despite these efforts, many people with disabilities continue to face barriers including physical and social barriers [3,18-24] that limit their use of PT.

Recently, Unsworth et al [20] examined, in their systematic review, the evidence that solely investigated the use of PT by mobility device users, which largely comprised people with mobility disabilities. The study identified problems encountered by mobility devices users, such as uneven pavement surfacing, lack of dropped curbs, steps instead of ramps, narrow doorways, controls for pedestrian lights or lift access that are too high, and badly designed street displays; problems with information being placed out of reading reach, inappropriate spaces for wheeled mobility devices, and lengthy wait times with little shelter [20]. Since the study excluded other types of disabilities such as visual; hearing; autism; and cognitive, mental, or intellectual disabilities, its findings cannot be generalized to all people with disabilities. Identification of needs related to PT of people with other types of disabilities is also important. For example, people with mental health conditions might not use certain services because of the existing social barriers, such as stigma [1], which differ from physical barriers commonly encountered by people with mobility impairment.

This study primarily aims to examine barriers and facilitators to the use of the PT (buses, trains, tramways, ferries) by people with different types of disabilities in the whole travel chain, where disability is defined as the interaction between individuals with a health condition (could be physical or mental) with personal and environmental factors including negative attitudes, inaccessible transportation and public buildings, and limited social support, etc [25]. For the purpose of this review, the definition includes older adults, as people with disabilities are aging with the increase of level of disabilities, and older adults commonly experience disabilities because of various age-related health conditions [26]. Second, this study aims to highlight the perceived experiences of individuals with disabilities when using PT in terms of their self-efficacy and satisfaction.

## Methods

### Design

A scoping review will be conducted using the PRISMA-ScR (Preferred Reporting Items for Systematic Reviews and Meta-Analyses Extension for Scoping Reviews) checklist [27]. The extent, range, and the nature of research activity will be explored to determine the value of undertaking a full systematic review, to summarize and disseminate research findings, and to identify research gaps in the existing literature [28]. For this scoping review, the methodological framework described by Arksey and O’Malley [28] will be used. It includes five stages: (1) identifying the research question; (2) identifying relevant studies; (3) study selection; (4) charting the data; and (5) collating, summarizing, and reporting the results. This last stage is described in the Results section.

### Identifying the Research Question

This phase involved a discussion among the authors (CRM, KLB, and FR) to identify the relevant research that arises from the gaps in the existing literature on the topic of this scoping review. The main research questions identified by the authors to guide the scoping review process were as follows: What are the different barriers and facilitators to the use of PT experienced by people with various types of disabilities including older persons? Second, what is their perceived experience in terms of satisfaction or self-efficacy when using PT? These questions emerge from a preliminary search of the literature using Google Scholar.

### Identifying Relevant Studies

An extensive literature search of both peer-reviewed and gray literature databases will be performed to identify relevant information about the topic. To conduct this research robustly, we will seek the collaboration of a librarian (MG) who specializes in the use of databases related to the rehabilitation and accessibility of PT by people with disabilities. Given that PT accessibility should consider several fields such as biomedical, transportation, psychological, and human and social sciences, the literature search will be conducted in 5 relevant databases including MEDLINE, Transport Database, PsycINFO (from Ovid platform), Embase, and Web of Science from 1995 to 2022. 1995 date was selected as the start date because very little literature was published in the field prior to this time [20]. The keywords and terms that will be used are as follows: (((Aged or ag?ing or old*) ADJ2 (person* or adult* or people or individual* or man or men or woman or women or user*)) or elder* or geriatric*).ti,ab,kw OR ((mental* or intellectual*) ADJ2 (retard* or deficien* or ill or illness)).ab,ti,kw OR ((physical* or hearing or visual* or cogniti*) ADJ2 (deficien* or impair* or challenge*)).ab,ti,kw OR (disabled or disabilit* or Handicap*or blind* or “hard of hearing” or deaf*).ab,ti,kw OR (((walking) ADJ2 (frame or stick)) or wheelchair* or scooter* or walker* or rollator* or crutche* or cane* or brace*).ti,ab,kw OR ((mobility or walking) adj2 (difficult* or limitation*)).ti,ab,kw AND (“Public transport* accessibility” or bus or buses or tramway* or train or trains or subway* or metro or underground or ferry or ferries).ti,ab,kw AND (satisfaction or “self-efficacy” or “self-efficacy” or confiden* or experience or “quality of life”).ti,ab,kw. Since the quality of life includes many aspects of life, such as psychological factors, we inserted this term just for optimizing the search function. This search function could be modified, if necessary, by switching from one database to another. A special mention has to be made for the Web of Science database. Indeed, we will activate the exact search from the advanced search to limit the lemmatization of the results. All retrieved literature will be imported into Covidence (Veritas Health Innovation), which is a software in which all the steps of a knowledge synthesis can be completed, where the duplicates will be removed from the selection of articles for the extraction of obtained data. The literature that meets the criteria presented in Textbox 1 will be included in our scoping review. Moreover, when a study has addressed the accessibility of multiple modes of PT, including fixed-route PT, it will be retained. However, only data from fixed-route PT will be considered in this study. In addition, when any systematic review related to the topic of this scoping review will be identified, we will count the number of studies included in the review that potentially meet our inclusion criteria and will note how many studies are not present in our search [27].

**Inclusion or exclusion criteria.**

**Inclusion criteria**

- Original peer-reviewed manuscript
- Guideline report
- Editorial
- Concerned with PT (public transport; bus, train, tramway, ferry) accessibility or confidence and satisfaction with PT for people with disabilities including older people
- Published from 1995 to 2022
- Published in English or French

**Exclusion criteria**

- Article for which the full text is not available
- Article focuses on the use of technology while using the PT system
- Article generally discusses universal design or transportation
- Validation study of measurement tools assessing PT accessibility or travel confidence or travel satisfaction for people with disabilities
- Study on no-fixed route PT accessibility (eg, adapted school bus for students with disabilities, paratransit)

### Study Selection

Two reviewers (CRM and SG) will independently select the literature to be included in the scoping review in the 2 steps of screening provided in the Covidence platform, based on the inclusion and exclusion criteria. The first step, after removing duplicates, will consist of screening the title and abstract for each document by examining how they are related to the topic. The second step will be reviewing the remaining full articles. All reasons for exclusion will be recorded in the Covidence platform, and cases of disagreement will be resolved through discussion between at least 2 reviewers. To prevent errors and reduce the risk of discrepancies between the 2 reviewers, each screening phase will start with a calibration exercise. In case of persistent disagreement, a third evaluator will be involved.

### Charting the Data

This stage involves the use of a data-charting form to extract relevant information from the reviewed literature [28]. For this scoping review, the data-charting form will be jointly developed by 2 reviewers to determine which variables to be extracted. The same reviewers (CRM and SG) will independently chart the data, discuss the results, and continuously update the data-charting form in an iterative process [29] by using Covidence. Information, such as author names and year of publication, setting of the study, study objective, sample, study design, intervention (if applicable), outcomes, and relevant findings to the research questions, will be extracted.

### Ethical Considerations

Research ethics approval is not required for this scoping review as the study will not include human or animal participants. Data will be sourced only from peer-reviewed and gray literature.

## Results

The search we performed on July 21, 2022, in the databases mentioned above allowed us to retrieve 6399 citations. Of these citations, 31 articles were identified, and data extraction was performed. As of March 11, 2023, we have started data analysis. Relevant findings related to barriers and facilitators, as well as the perceptions of people with disabilities in terms of self-efficacy and satisfaction when using PT, will be presented in tables. In addition, a narrative report will allow us to summarize the relevant findings from the key terms. For this purpose, a brainstorming session that involves all authors will be organized. The results will be described according to the Human Development Model-Disability Creation Process (HDM-DCP) conceptual framework [30] with respect to the research questions, and the aims of this scoping review. This conceptual framework addresses situations of disability that occur when personal and environmental factors restrict life habits, thereby decreasing social participation. Any gap or limitation in the existing literature will be identified and highlighted. We expect to complete this scoping review by June 2023.

## Discussion

### Expected Findings

This scoping review aims to examine the barriers and facilitators to the use of TP (buses, trains, tramways, and ferries) by people with various disabilities in the whole travel chain and highlight their perceived experiences in terms of satisfaction and self-effectiveness during their travel in PT. Since barriers and facilitators differ between people with disabilities, focusing on the literature on these indicators of PT use by people with various disabilities might help to provide recommendations for making PT more accessible and usable equitably by all passengers. Accessibility and use of PT ensure the participation and social inclusion of people with disabilities. In contrast, an inaccessible mode of PT will never be used by people with disabilities, and therefore may lead to their isolation and social exclusion. To better understand the issue of barriers and facilitators to the use of PT, Iwarsson and Ståhl [6] proposed the concept of the travel chain in PT. The travel chain suggests that any given travel starts at the origin of the users (eg, their home) and ends at the final destination. Thus, travel needs to be considered an indivisible whole rather than singling out each element. Jensen et al [31] underlined taking a sustained look at all the problems that people with disabilities might experience along an entire route, not simply considering the physical accessibility of PT vehicles. In this way, the elements of the travel chain mentioned above, including drivers’ and passengers’ attitudes, cannot be considered individually. For example, encountering a sidewalk lacking a curb cut for a wheelchair user is similar to a nondisabled person who encounters an impassable brick wall along their route to the bus stop. Minimally, such barriers can cause distress to the individual with a disability involved, and in the worst case, they can lead to journeys not being made [7]. Any barrier to all or part of the chain of PT use, and negative experiences, may affect the self-efficacy and satisfaction levels of people with disabilities, and therefore their willingness to travel. In return, these psychological factors may enhance travel behavior if PT is accessible and people with disabilities experience positive emotions throughout the travel.

This review will contribute evidence on the multiple barriers and facilitators that may be encountered by people with various forms of disabilities or in situations of autonomy loss (eg, older adults) throughout the fixed-route PT. Barriers and facilitators to PT may be experienced differently by people with disabilities depending on their individual situation of disability (eg, physical, intellectual, cognitive, visual, or hearing disability). It is therefore important to review the scientific literature that addresses PT issues to provide recommendations for best practices in PT to transport providers and policy makers to work together to make PT accessible, usable, and inclusive for people with all types of disabilities. Modifications to the environment (eg, installation of ramps) and interventions (eg, staff awareness and education, training in the use of PT for people with disabilities) may facilitate accessibility and use of PT by people with disabilities. Improved use of PT may develop access to the community and community-based services. Therefore, improved use of PT may facilitate social inclusion, participation, and well-being for people with disabilities.

### Limitations

This scoping review may exclude some studies. Indeed, the keywords to be used in the search strategy are broad and may not identify all specialized studies in PT accessibility for people with disabilities. Moreover, the fact of having considered only English and French as the languages of publication could exclude papers that are relevant for our scoping review.

### Conclusion

The identification of barriers and facilitators to the use of PT by people with disabilities is an important step that may help policy makers and transport operators around the world to develop and implement interventions to facilitate access, use, and inclusion of this mode of transport, as the experiences of people with disabilities when using this mode of transport have an impact on their well-being. The results of this scoping review could lead to a better understanding of the potential barriers and facilitators to the use of PT by people with various disabilities and how negative or positive experiences throughout the travel may influence their self-efficacy and satisfaction.

## Abbreviations

ADA: Americans with Disabilities Act
DDA: Disability Discrimination Act
HDM-DCP: Human Development Model-Disability Creation Process
PRISMA-ScR: Preferred Reporting Items for Systematic Reviews and Meta-Analyses Extension for Scoping Reviews
PT: public transport
UN-CRPD: United Nations Convention on the Rights of Persons with Disabilities
WHO: World Health Organization

## Data Availability

The data sets generated or analyzed during this study are available from The Open Science Framework (OSF; registration DOI: https://doi.org/10.17605/OSF.IO/2JDQS).
